# Body Fat Percentage Is More Strongly Associated With Handgrip Strength Than Body Mass Index Among North Indian Adults

**DOI:** 10.7759/cureus.111935

**Published:** 2026-07-02

**Authors:** Upendra Baitha, Mansi Sagwan, Shikhar Gupta, Ankush Garg, Gaurav Gupta, Arvind Kumar, Ranveer S Jadon, Prayas Sethi, Ashish D Upadhyay, Naval K Vikram, Naveet Wig, Piyush Ranjan

**Affiliations:** 1 Department of Medicine, All India Institute of Medical Sciences, New Delhi, New Delhi, IND; 2 Department of Gastroenterology, All India Institute of Medical Sciences, New Delhi, New Delhi, IND; 3 Department of Internal Medicine, All India Institute of Medical Sciences, Vijaypur, Jammu, Jammu, IND; 4 Department of Statistics, All India Institute of Medical Sciences, New Delhi, New Delhi, IND

**Keywords:** body composition, body mass index (bmi), handgrip strength, lower limb muscle strength, percentage body fat

## Abstract

Background

Muscle strength is a key factor determining metabolic health, physical performance, and morbidity. Although body mass index (BMI) is used widely to assess obesity, it does not assess body composition adequately. Percentage body fat (PBF) could provide a better estimate of adiposity and its association with muscle strength, particularly in Asian populations with higher body fat at lower BMI levels. We aimed to evaluate the associations of BMI and PBF with HGS and lower limb muscle strength among North Indian adults and to compare the independent associations of BMI and PBF with HGS.

Methodology

This cross-sectional study included 1,513 apparently healthy adults aged 18 to 60 years. Anthropometric measurements, BMI, and PBF were measured using bioelectrical impedance analysis. HGS was measured using a Jamar hydraulic dynamometer, and lower limb muscle strength was tested using a handheld dynamometer. Correlation and multiple regression analyses were performed to assess the association of BMI and PBF with muscle strength.

Results

The average age of the participants was 38.25 ± 9.76 years, and 54.5% were males. PBF showed a significant inverse association with HGS for both hands. For the right HGS, the regression coefficient B was −0.619 with an R² of 0.331, while for the left HGS, B was −0.594 with an R² of 0.336 (p < 0.001 for both). However, BMI showed a comparatively weaker association with HGS, explaining less than 2% of the variation. Higher BMI was significantly associated with greater lower limb muscle strength (p < 0.001), while no significant association was found between BMI categories and HGS. Multivariable regression analysis further showed that PBF remained independently associated with HGS (p-value < 0.001 for both hands).

Conclusions

According to our study, PBF demonstrated a stronger association with muscle strength than BMI. These findings show that relying on BMI alone is insufficient and suggest including body composition assessment to identify individuals at risk of reduced muscle strength and related health complications.

## Introduction

Muscle strength is defined as the maximum force a muscle group can generate against resistance, which serves as a critical indicator of physical function, metabolic health, and mortality. While BMI and body weight fluctuate, increased adiposity leads to intramuscular fat infiltration. This ectopic fat accumulation impairs contractile function, alters muscle architecture, and promotes localized chronic low-grade inflammation, thereby reducing both relative muscle strength and muscular endurance [1,2].

Assessment of handgrip strength (HGS) has emerged as a simple, reliable, and non-invasive technique for overall muscle strength in clinical and epidemiological settings [3]. Its ease of measurement, reproducibility, and significant impact on disability, metabolic disorders, and mortality strengthen its wide usage [4].

Thus, it is crucial to understand the determinants of muscle strength, especially modifiable factors such as body mass index (BMI) and percentage body fat (PBF). BMI is an extensively used indicator for classifying individuals based on weight relative to height and is commonly employed as a marker of body fat and nutritional status. However, its inability to distinguish between lean body mass and fat mass limits its capacity to reflect body composition adequately [5].

On the other hand, PBF may be a better indicator of adiposity with distinct physiological implications than only BMI. Although previous reports suggest that BMI may have a positive association with muscle strength, excess fat, especially central obesity, may negatively impact muscle quality and function [6].

There is a complex relationship between body fat, BMI, and muscle strength. Existing reports have indicated that individuals with higher BMI may have greater HGS due to increased muscle mass accompanied by higher body weight [7].

Conversely, studies incorporating PBF indicate that excess fat may impair muscle function through mechanisms such as intramuscular fat infiltration, chronic low-grade inflammation, and decreased muscle quality, even among individuals with normal BMI; this phenomenon is called “normal weight obesity” [6,8]. As India is undergoing a rapid nutritional and epidemiological transition characterized by a dual burden of undernutrition and an increasing prevalence of overweight and obesity, it is imperative to study these associations. Additionally, South Asian individuals have higher PBF at lower BMI levels than individuals in Western populations, leading to metabolic and functional impairments despite a normal BMI [5,9]. Previous Indian studies have reported significant associations between PBF, BMI, and HGS, suggesting that excess fat and undernutrition can significantly affect muscle strength [10]. However, there is a paucity of large-scale studies estimating these associations in adult populations, especially in North India.

Muscle strength is a significant predictor of long-term health outcomes. Reduced HGS is associated with an increased risk of cardiometabolic disorders such as diabetes, hypertension, psychological health, and impaired cognitive function [4,11,12]. With the rising incidence of non-communicable diseases in India, early identification of indicators such as reduced muscle strength and its relationship with modifiable parameters is crucial for preventive health strategies.

This study aims to evaluate the associations of BMI and PBF with HGS and lower limb muscle strength among North Indian adults and to compare the independent associations of BMI and PBF with HGS. With the simultaneous evaluation of anthropometric and body composition parameters, the study seeks to generate a more comprehensive understanding of how body composition influences muscle strength. The findings may help inform the assessment of adiposity and muscle strength in North Indian adults and provide a basis for future research.

## Materials and methods

**Figure 1:**
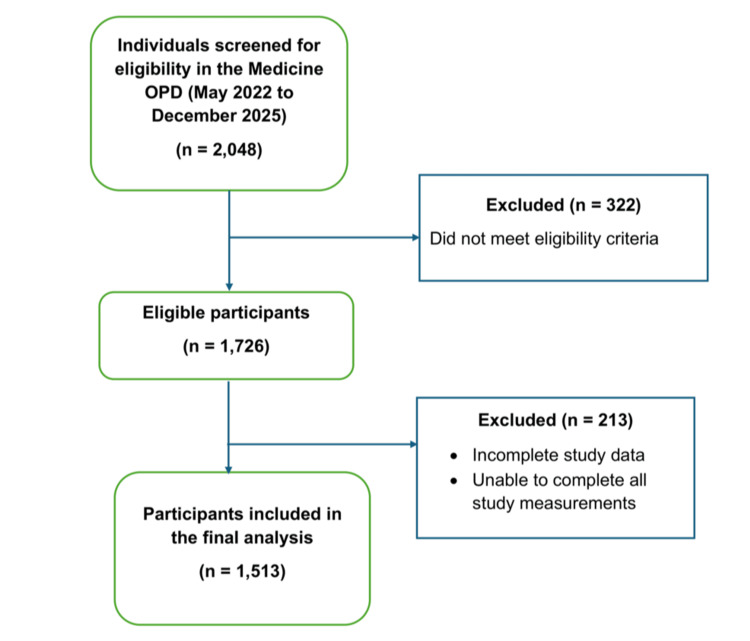
Participant selection flow diagram depicting the enrollment of participants for the study.

We conducted an analytical cross-sectional study in the Department of Medicine at the All India Institute of Medical Sciences (AIIMS), New Delhi, over a period of three years from May 2022 to December 2025. The study comprised 1,513 participants aged 18 to 60 years. The study employed a hospital-based convenience sampling method. Apparently healthy participants who were visiting the Medicine OPD at AIIMS, New Delhi, during the study period were enrolled in the study if found eligible as per the inclusion and exclusion criteria (Figure 1).

Inclusion and exclusion criteria

Apparently healthy adults between the age range of 18 and 60 who willingly provided written informed consent were included in the study. Individuals with serious medical conditions, including severe heart conditions, pulmonary conditions, chronic kidney disease, chronic liver disease, and neuromuscular junction diseases, were excluded. People with any acute illnesses in the previous three months and trauma or significant surgery within the previous six months were also excluded.

Methods and procedure

Anthropometric Parameters and Body Mass Index

An electronic scale was used to assess body weight, and a fixed stadiometer was used to measure body height with subjects dressed in light clothing and barefoot. After obtaining informed consent and meeting the inclusion criteria, the participants were enrolled. We classified participants into the following four BMI categories for analysis according to the Asia-Pacific recommendation criteria for Indians: <18.5 kg/m² was considered underweight, 18.5-22.9 kg/m² was classified as normal, 23-24.9 kg/m² was categorized as overweight, and ≥ 25 kg/m² was categorized as obesity [13]. BMI was calculated using Quetelet’s index (BMI = weight in kg/height in m²) [14].

Percentage Body Fat

PBF was determined using a handheld bioelectrical impedance analysis (BIA), which is used to measure body composition. The ACCUNIQ BC720 Body Composition Analyzer, manufactured by SELVAS Healthcare (Republic of Korea), was used. The analyzer comprises a multi-frequency BIA technique based on a tetrapolar electrode system with 8-12 touch electrodes. Calibration was performed under the Weight/Height menu in the system setup. Before the evaluation, subjects were advised to refrain from consuming alcohol, engaging in intense physical activity, and consuming caffeine-containing beverages or diuretics for four hours before the test. The measurement was performed in the morning after an overnight fast. Before the assessment, the subjects were asked to remove shoes, socks, metallic accessories, and any items that may affect electrical conductivity. Hands and feet were clean and dry. Subject details, including UHID, age, sex, and height, were entered into the ACCUNIQ analyzer, and electrode plates were checked for cleanliness. The subject stood barefoot on the foot electrodes with proper heel and forefoot contact and firmly held the hand electrodes, ensuring full contact of the palms, fingers, and thumbs. During measurement, the arms were abducted approximately 45° from the body, and the legs were kept apart to avoid contact. The subject remained still, maintained the posture, and refrained from talking throughout the test for about a minute. The measurement was stopped if all eight electrodes were not in proper contact. The ACCUNIQ analyzer uses a safe, low-level electrical current at multiple frequencies to measure impedance and estimate body composition. The analysis was completed within approximately 30-60 seconds, generating the body composition report.

Measurement of Handgrip Strength and Muscle Strength

HGS was measured using Jamar’s hand-held hydraulic dynamometer. The participants were asked to sit with their elbows bent and their hips and knees at a 90-degree angle on a typical straight-back chair without armrests. All participants were directed to squeeze the handle on verbal cue, with ongoing verbal encouragement not exceeding 10 seconds from the healthcare personnel. To minimize muscle fatigue and ensure maximum voluntary contraction, three readings were taken from each hand, with a rest period of 30 seconds, and the mean of the three readings was used as the representative HGS for each hand. This method, used extensively in previous literature, improves reliability and reduces measurement variability [15,16]. The Lafayette hand-held dynamometer (Model 01165A, Lafayette Instrument Company, Indiana, USA) was used to measure the bilateral strength of lower limb muscle groups, including hip flexors, knee flexors, knee extensors, and ankle dorsiflexors, and the value was recorded in kilograms (kg) for analysis. Testing was performed by a single, trained examiner to maintain consistency. Participants were made to sit on an examination table with their hips and knees fixed at 90 degrees, with standardized positions used for each muscle group. The examiner applied isometric resistance perpendicular to the limb segment. Each contraction was maintained for five seconds with a 30-second rest period between trials. The highest peak force value generated across three trials was recorded and used for analysis.

Ethics review

The study was reviewed and approved by the Ethics Committee, AIIMS, New Delhi. Written informed consent was obtained from all subjects, and only those willing to participate were included in the study. Prior approval was acquired from the Institutional Ethical Committee (approval number: IEC-329/01.04.2022). Confidentiality was maintained with the collected data, and the study was conducted with the utmost respect for ethical standards while ensuring participant well-being.

Statistical analysis

Data for the study were collected using electronic methods and manually checked for errors. Reference values were created for each age and sex group using STATA 16 software (StataCorp., College Station, TX, USA). Data normality was tested with the Kolmogorov-Smirnov test. Independent-samples t-tests were used to compare strength and flexibility between males and females. Multiple regression analysis was performed to examine how sex and body measurements, i.e., BMI and body composition, affect muscle strength and joint flexibility. The data were summarized using averages, means, standard deviations, and proportions. The Pearson correlation coefficient (r) was used to measure the strength of relationships between variables. Continuous data was also analyzed using scatter plots and regression to understand patterns and measure effects. One-way analysis of variance (ANOVA) followed by multiple comparisons using the Bonferroni test was performed. A p-value ≤0.05 was considered statistically significant.

## Results

The study included 1,513 participants, including 824 (54.5%) males and 689 (45.5%) females. The mean age of our study participants was 38.25 ± 9.76 years. The mean BMI was found to be 27.83 ± 4.72 kg/m². On average, participants were classified as overweight according to the Asia-specific criteria. A higher mean BMI was found in female participants when compared to males (p < 0.001). The mean PBF was found to be 35.44 ± 9.15%, with a marked difference between genders, as females had a significantly higher PBF than males (p < 0.001). The mean HGS was significantly higher in males compared to females in both hands (p < 0.001) (Table 1).

**Table 1:** Comparison of demographic characteristic body composition and HGS between males and females. Values are expressed as mean ± standard deviation (SD) *: Independent-samples t-test. BMI = body mass index; HGS = handgrip strength; PBF = percentage body fat

Parameters	N = 1,513	Mean	SD	t-value (df = 1,511)	P-value*
Age (years)					
Male	824	38.45	10.38	-0.83	0.40
Female	689	38.02	8.96		
Total	1513	38.25	9.76		
BMI (kg/m²)					
Male	824	26.86	4.38	8.67	<0.001
Female	689	28.96	4.86		
Total	1513	27.83	4.72		
PBF (%)					
Male	824	29.9	7.02	34.21	<0.001
Female	689	42.05	6.70		
Total	1513	35.44	9.15		
Right HGS (kg)					
Male	824	39.84	7.67	41.40	<0.001
Female	689	25.41	5.43		
Total	1513	33.27	9.85		
Left HGS (kg)					
Male	824	37.71	7.54	39.93	<0.001
Female	689	24.19	5.13		
Total	1513	31.55	9.39		

In our study population, males showed a higher mean muscle strength in all lower limb parameters compared to females, and this also achieved a statistically significant difference (p < 0.001) (Table 2).

**Table 2:** Comparison of muscle strength of lower limb between males and females. Values are expressed as mean ± standard deviation (SD). *: Independent-samples t-test.

Muscle strength of the lower limb (kg)	Total (mean ± SD) (N = 1,513)	Males (mean ± SD) (N = 824)	Females (mean ± SD) (N = 689)	t-value (df = 1,511)	P-value*
Right hip joint flexors	8 ± 1.18	8.57 ± 1.05	7.31 ± 0.94	-24.34	<0.001
Left hip joint flexors	7.91 ± 1.18	8.46 ± 1.06	7.24 ± 0.97	-23.03	<0.001
Right knee joint extensors	8.36 ± 2.12	8.84 ± 2.56	7.78 ± 1.20	-9.95	<0.001
Left knee joint extensor	8.25 ± 2.76	8.74 ± 3.49	7.67 ± 1.23	-7.65	<0.001
Right knee joint flexors	7.5 ± 1.15	7.93 ± 1.08	6.97 ± 0.99	-17.81	<0.001
Left knee joint flexors	7.36 ± 1.14	7.79 ± 1.09	6.85 ± 0.96	-17.53	<0.001
Right ankle joint dorsiflexion	6.77 ± 1.29	7.19 ± 1.29	6.27 ± 1.10	-14.66	<0.001
Left ankle joint dorsiflexion	6.72 ± 1.33	7.14 ± 1.33	6.22 ± 1.16	-14.21	<0.001

For assessing the effect of BMI on HGS, the participants were categorized into four BMI groups (underweight, normal, overweight, and obese), and HGS was compared across these groups. We did not observe a statistically significant difference in HGS across the BMI categories. However, we observed a contrasting trend among the muscle strength parameters of the lower limbs, which achieved a statistically significant increase with higher BMI categories (p < 0.001) (Table 3).

**Table 3:** Comparison of HGS and lower limb muscle strength across different BMI categories. Values are expressed as mean ± standard deviation (SD). *: One-way ANOVA test. BMI = body mass index; HGS = handgrip strength; ANOVA = analysis of variance

BMI categories (N = 1,513)	Underweight (N = 33)	Normal (N = 168)	Overweight (N = 202)	Obese (N = 1,110)	F value (df = 3, 1,509)	P-value*
Right HGS (kg)	33.54 ± 8.77	33.96 ± 9.68	33.89 ± 9.23	33.05 ± 10.02	0.74	0.53
Left HGS (kg)	31.67 ± 8.43	32.39 ± 9.13	32.36 ± 8.93	31.27 ± 9.53	1.28	0.28
Muscle strength of the lower limb (kg)						
Right hip joint flexors	7.015 ± 1.33	7.80 ± 1.26	7.99 ± 1.28	8.06 ± 1.13	10.37	<0.001
Left hip joint flexors	6.85 ± 1.37	7.61 ± 1.24	7.90 ± 1.30	7.98 ± 1.13	14.00	<0.001
Right knee joint extensors	7.19 ± 1.57	7.82 ± 1.50	8.17 ± 1.57	8.51 ± 2.27	9.55	<0.001
Left knee joint extensor	6.88 ± 1.55	7.58 ± 1.52	8.05 ± 1.61	8.43 ± 3.05	7.95	<0.001
Right knee joint flexors	6.51 ± 1.03	7.18 ± 1.17	7.38 ± 1.20	7.59 ± 1.11	16.02	<0.001
Left knee joint flexors	6.44 ± 1.02	7.11 ± 1.20	7.26 ± 1.20	7.45 ± 1.104	12.95	<0.001
Right ankle joint dorsiflexion	5.35 ± 0.855	6.27 ± 1.401	6.54 ± 1.26	6.94 ± 1.24	31.49	<0.001
Left ankle joint dorsiflexion	5.28 ± 1	6.19 ± 1.40	6.48 ± 1.34	6.89 ± 1.28	31.24	<0.001

**Figure 2:**
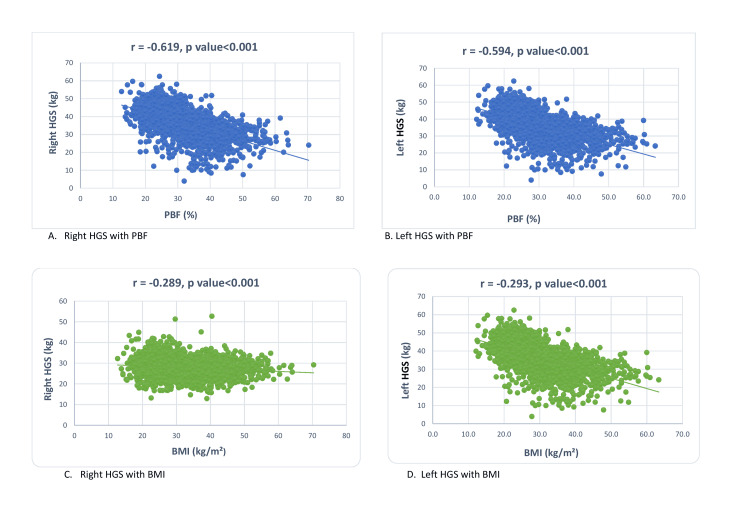
Correlation of HGS with PBF and BMI using Karl Pearson’s correlation test (N = 1,513). (A) Right HGS with PBF. (B) Left HGS with PBF. (C) Right HGS with BMI. (D) Left HGS with BMI. BMI = body mass index; HGS = handgrip strength; PBF = percentage body fat

We assessed the association of HGS with PBF. PBF showed a significant inverse association with HGS for both hands. For the right HGS, the regression coefficient (B) was -0.619 with an R² of 0.331 (p < 0.001). For the left HGS, the regression coefficient (B) was -0.594, with an R² of 0.336 (p < 0.001). BMI showed a statistically significant but comparatively weaker association with HGS than PBF. For the right HGS, the regression coefficient (B) was -0.289, with an R² of 0.019 (p < 0.001), and for the left HGS, the regression coefficient (B) was -0.293, with an R² of 0.021 (p < 0.001) (Table 4, Figure 2).

**Table 4:** Univariate linear regression analysis of the association of PBF and BMI with HGS (N = 1,513). *: Linear regression  analysis with predictor/independent variables: PBF (%) and BMI (kg/m²) separately. Outcome/dependent variable: HGS (kg). R² indicates the proportion of variance explained by the regression model. BMI = body mass index; HGS = handgrip strength; PBF = percentage body fat; CI = confidence interval

Independent predictors/variables	Model significance	B coefficient (95% CI)	R^2^	P-value*
Right HGS				
PBF	F (1, 1,511) = 748.27	-0.619 (-0.663 to -0.575)	0.331	<0.001
BMI	F (1, 1,511) = 29.29	-0.289 (-0.394 to -0.184)	0.019	<0.001
Left HGS				
PBF	F (1, 1,511) = 765.25	-0.594 (-0.636 to -0.552)	0.336	<0.001
BMI	F (1, 1,511) = 33.21	-0.293 (-0.393 to -0.193)	0.021	<0.001

A multivariable regression model was constructed to assess the association of BMI and PBF with HGS after adjustment for demographic and lifestyle factors. PBF was significantly and inversely associated with both hands (p < 0.001). BMI was positively associated with right HGS but showed no significant association with left HGS (p = 0.11) (Table 5).

**Table 5:** Multivariable linear regression analysis of the association between PBF, BMI, and HGS after adjusting for potential confounders. *: Multivariable linear regression analysis adjusted for covariates. Adjusted R² indicates the proportion of variance in HGS explained by the model. Independent variables of interest were PBF (%) and BMI (kg/m²). Models were adjusted for age, sex, and other covariates included in the regression model; outcome/dependent variable: HGS (kg). BMI = body mass index; HGS = handgrip strength; PBF = percentage body fat; CI = confidence interval

Variables	Regression coefficient (B)	Standard error	95% CI	F-value (df = 15, 1,497)	Adjusted R²	P-value*
Right HGS						
PBF	-0.14	0.02	-0.18 to -0.09	F (15, 1,497) = 131.81	0.57	<0.001
BMI	0.08	0.03	0.01 to 0.16	F (15, 1,497) = 127.94	0.55	<0.001
Left HGS						
PBF	-0.15	0.02	-0.20 to -0.10	F (15, 1,497) = 127.00	0.56	<0.001
BMI	0.05	0.03	-0.01 to 0.12	F (15, 1,497) = 121.15	0.54	0.11

## Discussion

In this study, we measured the participants’ HGS, lower limb muscle strength, PBF, and BMI. We also looked at how body fat and BMI influence muscle performance and whether both have similar effects or not. For a long time, there has not been a clear “gold standard” for diagnosing obesity. Because of its ease of measurement, BMI has become the most commonly used method. However, many studies highlight that BMI should be used carefully. The cut-off values for BMI are adequate at confirming obesity when it is present, but not very effective at detecting all cases. This means it can miss people who actually have high body fat, in both children and adults, which could cause health problems over time [17,18].

The mean BMI of the subjects in our study was found to be 27.83 ± 4.72 kg/m², which is considerably higher, and achieved a statistically significant difference. However, in previous studies, the mean BMI was found to be lower, as reported by Shrestha et al., who reported a mean BMI of 20.76 ± 2.71 kg/m², while Al-Asadi reported a mean BMI of 23.82 ± 2.73 kg/m² [19,20]. Previously cited literature has shown a decline in grip strength with an increase in BMI, but our study found no association, and it did not achieve statistical significance [7].

The mean PBF of the participants in our study was found to be 35.44 ± 9.15%, which was significantly higher in females (females = 42.05% vs. males = 29.9%). This finding is consistent with existing literature, as studies conducted in the Indian population showed a PBF of 22.09% in males and 30.61% to 33.6% in females [1,21]. Similarly, studies from other parts of the world have reported findings that are consistent with our study. A broad study with more than 5,000 participants in China found PBF to be 25.74% in males and 34.01% in females [2]. Another study among young adults in Europe reported average body fat levels of 24.16% in males and 32.15% in females [22].

The mean HGS of the participants in both hands was similar to the findings reported by other authors, such as Shrestha et al. (34.24 ± 2.58 kg), Al-Asadi (34.1 ± 11.9 kg), and Lad et al. (33.33 ± 2.58 kg) [10,19,20]. However, it was much higher than the findings of Prakash et al., who reported a mean HGS of 26.51 ± 0.75 kg [23]. In an Australian population, Carmelli and Reed reported a much higher mean HGS of 47 ± 9.5 kg [24]. In our study, men showed significantly greater muscle strength in the lower limbs than women, likely due to their greater muscle mass, which contributed to increased muscle strength [3,5].

In comparison with BMI categories, our study did not show any significant association with HGS of both hands. This finding differs from previous literature. The study conducted by Lad et al. showed a positive association between HGS and BMI [10]. However, BMI is a crude measure that does not differentiate lean mass and fat mass; thus, it may be inaccurate in reflecting functional muscle strength [9]. BMI showed a significant positive association with lower limb parameters, which is similar to findings from previous studies, where greater body weight was associated with increased absolute strength in lower limbs due to chronic mechanical loading [7].

However, PBF exhibited a strong inverse association with HGS, explaining about one-third of its variability (R² = 0.331). Mechanisms such as fat build-up in muscles, physical inactivity, and increased inflammation may explain this association. All these adversely affect muscle quality and may lead to reduced muscle strength [6]. Although our findings are supported by existing studies, contrastingly, Gale et al. reported a positive correlation between PBF and HGS [25].

BMI showed a statistically significant inverse association with HGS; however, the association was substantially weaker than that observed for PBF, with BMI explaining less than 2% of variability, which highlights its limited use as an independent marker. After adjusting for potential confounding variables in multivariate regression analysis, PBF remained significantly associated with lower HGS, while BMI showed little or no independent association, suggesting that PBF may provide a better measure of body composition with regard to muscle strength than BMI alone. The emergence of PBF as a major contributor is consistent with findings from large epidemiological studies such as the PURE study [5]. The inverse association between PBF and HGS is also consistent with previous literature linking excess adiposity with impaired muscle quality and function [6,8]. As reduced HGS has the potential to predict cardiometabolic and cognitive risks, early identification is crucial [5,11,12].

Although we performed our study in a large cross-sectional dataset with 1,513 participants and included both upper and lower limb strength, it had certain limitations. The cross-sectional study design limits the ability to establish causal relationships between muscle strength and body composition parameters. As all eligible participants during the study period were included, a formal sample size calculation was not performed. Although BIA is a practical and widely used procedure in clinical settings, it estimates body composition indirectly and does not directly assess muscle quality, intramuscular fat infiltration, absolute lean mass distribution, or genetic and physiological determinants of muscle function. It may be less accurate compared to dual-energy X-ray absorptiometry and could be influenced by participant hydration status, recent food intake, physical activity, time of assessment, and ambient temperature [5]. The distribution of our sample was also highly skewed, with a large predominance of obese individuals (n = 1,110) and a very small underweight subgroup (n = 33), which may reduce the statistical power of subgroup analysis across BMI extremes. Although we adjusted for confounders such as gender, age, exercise/physical activity, occupation, smoking history, and alcohol consumption, other confounders such as dietary intake, comorbidities, and medication use could not be measured, which could have influenced muscle strength. Our study population consisted of individuals from the Medicine OPD of a tertiary care center in North India, which inherently introduces selection bias (Berkson’s bias) and limits the generalizability of our findings. Community-based studies are warranted to validate these findings. We assessed muscle strength using HGS and selected lower limb parameters, which, though widely accepted, may not fully assess overall muscle function. Although we measured the lower limb muscle strength, regression analyses to examine its independent association with PBF were not performed. Future studies should evaluate this relationship using dedicated multivariable analytical models.

## Conclusions

The present study demonstrated a significant inverse association between PBF and muscle strength, with PBF showing a stronger association with HGS than BMI in our study population. These findings suggest that assessment of body composition may provide additional information beyond BMI alone for identifying individuals with excess adiposity who may be at risk of reduced muscle strength. This may be particularly relevant in South Asian populations, who tend to have higher body fat percentages at lower BMI levels. Incorporating body composition assessment into routine clinical and public health evaluations may facilitate earlier identification of individuals at increased risk of adverse metabolic and functional outcomes.
